# Disruption of Structural Disulfides of Coagulation FXIII-B Subunit; Functional Implications for a Rare Bleeding Disorder

**DOI:** 10.3390/ijms20081956

**Published:** 2019-04-22

**Authors:** Sneha Singh, Mohammad Suhail Akhter, Johannes Dodt, Amit Sharma, Senthilvelrajan Kaniyappan, Hamideh Yadegari, Vytautas Ivaskevicius, Johannes Oldenburg, Arijit Biswas

**Affiliations:** 1 Institute of Experimental Haematology and Transfusion Medicine, University Clinic Bonn, 53127 Bonn, Germany; sneha.gupta@ukbonn.de (S.S.); suhailaiims@gmail.com (M.S.A.); sharmaaiims@gmail.com (A.S.); hamideh.yadegari@ukbonn.de (H.Y.); vytautas.ivaskevicius@ukbonn.de (V.I.); johannes.oldenburg@ukbonn.de (J.O.); 2 College of Applied Medical Sciences, Jazan University, Jizan 82911, Saudi Arabia; 3 Paul-Ehrlich-Institute, 63225 Langen, Germany; johannes.dodt@pei.de; 4 Department of Hematology, All India Institute of Medical Sciences, New Delhi 110029, India; 5 DZNE, German Center for Neurodegenerative Diseases, 53127 Bonn, Germany; Senthil.Kaniyappan@dzne.de

**Keywords:** coagulation Factor XIII, FXIII-B, disulfide bonds, FXIII deficiency

## Abstract

Congenital FXIII deficiency is a rare bleeding disorder in which mutations are detected in *F13A1* and *F13B* genes that express the two subunits of coagulation FXIII, the catalytic FXIII-A, and protective FXIII-B. Mutations in FXIII-B subunit are considerably rarer compared to FXIII-A. Three mutations in the *F13B* gene have been reported on its structural disulfide bonds. In the present study, we investigate the structural and functional importance of all 20 structural disulfide bonds in FXIII-B subunit. All disulfide bonds were ablated by individually mutating one of its contributory cysteine’s, and these variants were transiently expressed in *HEK293t* cell lines. The expression products were studied for stability, secretion, the effect on oligomeric state, and on FXIII-A activation. The structural flexibility of these disulfide bonds was studied using classical MD simulation performed on a FXIII-B subunit monomer model. All 20 FXIII-B were found to be important for the secretion and stability of the protein since ablation of any of these led to a secretion deficit. However, the degree of effect that the disruption of disulfide bond had on the protein differed between individual disulfide bonds reflecting a functional hierarchy/diversity within these disulfide bonds.

## 1. Introduction

Coagulation Factor XIII (FXIII) is a pro-transglutaminase that acts at the terminal stage of the blood coagulation cascade and is responsible for covalent cross-linking of pre-formed fibrin polymers making them resistant to premature fibrinolysis [1]. In plasma, FXIII exists as a zymogenic heterotetramer with non-covalently associated dimers of its catalytic FXIII-A and non-catalytic/carrier FXIII-B subunits. Thrombin activates this zymogenic complex by cleaving an N-terminal 37 amino acid long region on the FXIII-A subunit called the activation peptide (FXIII-AP). This cleavage is also accompanied by binding of calcium ions to FXIII-A, resulting in conformational changes that trigger the dissociation of the FXIII-B subunit as well as the opening up of the closed zymogenic dimeric form of the FXIII-A subunit into an open monomeric form of FXIII-A (FXIIIAa) facilitating substrate access to it catalytic site (a triad with a catalytic cysteine, i.e., Cys314 as its functional centre) [2]. Since this protein contributes to the stability of fibrin clots, inherited or acquired defects result in a bleeding predisposition [1]. The inherited form of FXIII deficiency is a rare coagulation disorder (one in one–four million) resulting from homozygous or compound heterozygous mutations in FXIII genes, and it usually causes a severe bleeding diathesis with umbilical cord bleeding as the most common symptom associated with this deficiency. More than 120 mutations have been detected in *F13A1* and *F13B* genes corresponding to the two subunits of FXIII since the first case was reported in 1962 by Duckert et al. [3,4,5]. More than 95% of the mutations in severe inherited FXIII deficiency occur in the *F13A1* gene (OMIM # 613225), but only a few mutations have been detected in the *F13B* gene (OMIM #613235) [5,6,7,8,9,10,11,12]. However, in the past decade, several reports from our group have indicated that the heterozygous form of this defect might also have clinical relevance as a mild form of FXIII deficiency, which we anticipate has a higher prevalence than severe inherited forms [13,14,15]. We have reported several mutations which were detected in individuals who suffer from inherited mild FXIII deficiency [14]. Many of these individuals are asymptomatic, but some also display unusual bleeding tendency when exposed to some kind of trauma. Interestingly, unlike the severe inherited form, in the mild form, the proportion of mutations detected in the FXIII-B subunit is far higher. Almost 20%–40% of the mutations detected in mild FXIII deficiency occur in *F13B* gene [5]. The catalytic FXIII-A subunit is a structurally and functionally well-characterized protein. Its partner FXIII-B subunit comparatively is a relatively unknown entity. There exists no biophysical structure for this protein, although based on its strong homology to complement factor H, several high-quality models of its repetitive sushi domains have been reported [16]. The FXIII-B protein is a traditionally secreted protein (bearing an N-terminal 20 amino acid long signal peptide) expressed in hepatocytes [17,18,19]. It associates with the FXIII-A subunit in the plasma to form the heterotetrameric complex. Since it is secreted in excess of FXIII-A subunit, it is also present in plasma in it’s unbound, free-form, which hints towards pleiotropic roles of this protein beyond coagulation [20]. Its circulating form had earlier been reported to be a monomer based on its sedimentation coefficient, although gel filtration results of FXIII-B expressed in insect cell lines indicate that it is a dimer [17]. Homology studies suggest that a monomer of FXIII-B subunit is composed of 10 repetitive sushi domains, held together by short peptide linkers. Sushi domains are also known as complement control modules since they also exist in complement system proteins like complement factor H (CFH) [21]. Functionally they act as chaperones to other catalytic proteins and regulate their functional states by binding to them. Each sushi domain has a conserved core structure with four consensus cysteine residues forming two disulfide bonds [22]. Therefore, a FXIII-B monomer will comprise of 20 disulfide bonds. The symmetrical arrangement of cysteine bond formation (abab pattern) in individual sushi domain gives specific intrinsic topology, thereby adopting a signature secondary structure, a β-sandwich type fold, and an overall globular shape. The disulfide bridges enable sushi domains to folds into a compact hydrophobic core enclosed by 3 + 2 beta-strands. Rigorous analysis of FXIII activation has revealed that the rate of activation of FXIII-A subunit is accelerated in the presence of FXIII-B subunit, which raises interest in its suggestive role in the regulation of FXIII-A mediated fibrin cross-linking [2,23]. Additionally, Souri et al., have suggested that FXIII-B mediates association of Fibrinogen, FXIII-A, and Thrombin, hence enhancing the cross-linking [23,24]. These developments in the last few years indicate that the role of FXIII-B in fibrin cross-linking extends beyond being a mere carrier/protective protein. Interestingly, three of the mutations detected in the FXIII-B subunit causing either severe or mild inherited FXIII deficiency, occur on the cysteines forming the structural disulfide bonds [5,13,14]. Transient expression of some of these mutations suggested pathomolecular influences on the core fold of the protein, consequently affecting its secretion. Disulfide bonds, structural or allosteric, play a major role in several coagulation proteins [25]. The disulfide bonds observed in FXIII-B are very likely structural, although no structural data exists for this subunit to confirm this assumption. The only structural data currently in literature comprises of electron microscopy studies which indicate the subunit to be filamentous in nature [26]. Even though the disulfide bonds present on each sushi domain of this subunit might be structural in nature, its relative contribution to the functional aspects of the protein might differ. Some functional data exists of the involvement of select sushi domains of the FXIII-B subunit to its overall fold (its dimeric form) or in the interaction with partner FXIII-A subunit [17,27].

In the present study, we investigate the structural and functional relationship of these disulfide bonds to the overall functionality of the protein. We ablate these disulfide bonds by mutating one contributory cysteine at a time and transiently express the resulting variant in HEK293t cell lines in order to study their effect on stability/secretion of the protein. We also purified the expressed variants to study the impact of these mutants on their mutual dimerization and on the activation of the FXIII-A subunit. In the absence of a biophysical structure, we have generated a monomeric FXIII-B subunit model by assembling high quality threaded models of its individual sushi domains. The structural flexibility of the different disulfide bonds within this model is subsequently investigated using classical unbiased all-atomic molecular dynamic MD simulation. We observed that although mutating specific disulfide bonds have differing degrees of functional effect on the protein based on which domain they belong to, almost all of them uniformly hinder the global fold of the protein, resulting in secretion defects. This explains earlier similar observations for FXIII-B subunit mutations detected in FXIII deficiency [14,16].

## 2. Results

### 2.1. Disruption of FXIII-B Subunit Structural Disulfides Results in a Secretion Deficit

**Figure 1:**
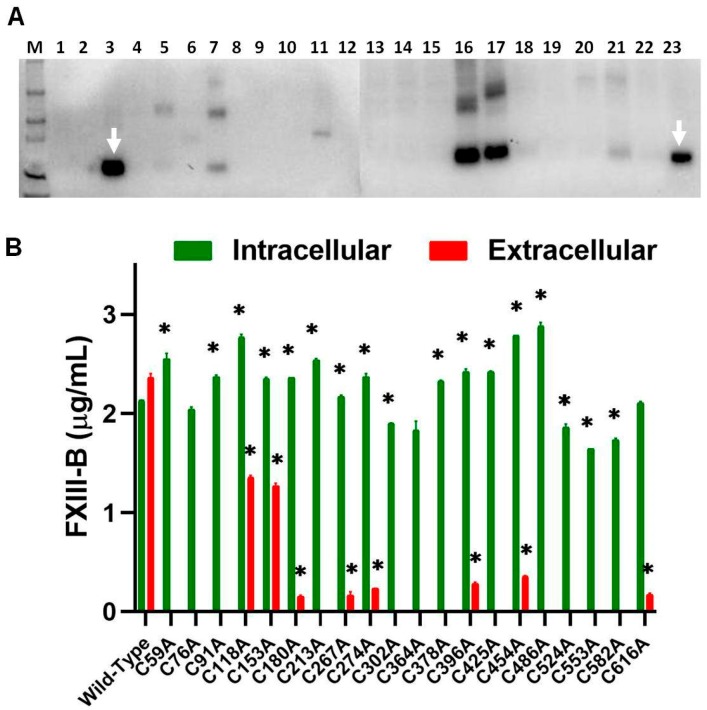
Transient expression of FXIII-B Cysteine mutants. **Panel A.** A conventional western blot of 10uL each of the wild type FXIII-B and 20 rFXIII-B cysteine mutants retained from the culture medium of transfected cells, probed against mouse to human FXIII-B antibody. **Lanes:** 1: Un-transfected control; 2: C76A; 3: Wild-Type; 4: C364A; 5: C396A; 6: C425A; 7: C454A; 8: C524A; 9: C553A; 10: C582A; 11: C616A; 12: C486A; 13: C378A; 14: C59A; 15: C91A; 16: C118A; 17: C153A; 18: C180A; 19: C213A; 20:C267A; 21:C274A; 22: C302A; and 23: rFXIII-B (Zedira, 75ng, positive control) White arrows represent the wild-type FXIII-B. **Panel B.** A comparative bar-plot representation of the antigenic levels of FXIII-B cysteine mutants versus the FXIII-B wild type evaluated on a quantitative sandwich ELISA based platform, detecting FXIII-B in 100µL of sample retained from transiently transfected cells. Green and red bars represent the intracellular and the extracellular fractions, respectively. A “*” symbol represents significance (*p*-value < 0.05).

All 20 FXIII-B cysteine mutants were successfully expressed intracellularly, but only 8 of these (C118A, C153A, C180A, C267A, C274A, C396A, C454A, and C616A) were successfully secreted out of the cell at levels detectable by ELISA and Western blot (Figure 1A,B). Intracellularly, except for C302A, C524A, C553A, and C582A variants, all other mutants were detected at levels similar to or greater than the wild type. The eight mutants that were successfully secreted showed significantly lower secreted protein than the wild type (Figure 1A). Among the three variants reported so far on these disulfide bonds, only one was earlier shown to secrete any detectable amounts (~30–50% of wild type) of protein, i.e., C336F in earlier expression based studies (C316A based on earlier nomenclature) [14]. In our study, we have mutated the oppositely paired cysteine to the C336, i.e., C378 to an alanine. The variant in our study unlike the reported mutant C336F is not secreted at all. Since the expression methodology, as well as the evaluation strategy of this study, is exactly the same as the expression study on C336F, we believe that the type of substitution occurring on the particular cysteine does influence the fate of the protein even if both substitutions result in disruption of the same disulfide bond. Very clearly, the alanine substitution on the oppositely paired cysteine results in far greater misfolding compared to the C336F variant. Therefore, while C336F also disrupts the same disulfide bond, the protein variant does manage to fold itself and get secreted out, albeit with much lower efficiency than the wild type.

### 2.2. Disruption of FXIII-B Subunit Structural Disulfides Results Mainly in ER Accumulation

**Figure 2:**
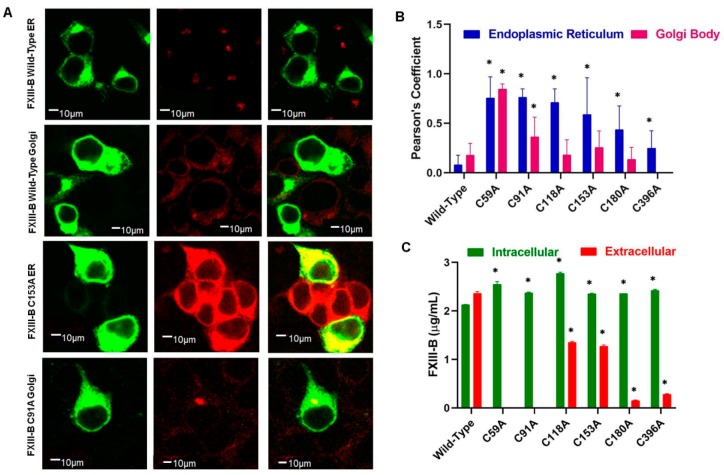
Effect of FXIII-B cysteine mutations on intracellular trafficking of FXIII-B protein. **Panel A.** Confocal microscopy tracking the subcellular localization of FXIII-B cysteine mutant proteins, via cell-specific markers; green (αFXIII-B), red (α-PDI (for endoplasmic reticulum) and α-TGN-46 (for trans-Golgi network)) with secondary antibodies conjugated with Alexa-488 and Alexa-555 respectively. Bars represent 10µm scale. **Panel B**. Bar-plot representation of Pearson’s coefficient calculated as a measurement of the intensity of pixels, defining co-localization of FXIII-B within either endoplasmic reticulum (PDI), or trans-GGolgi network (TGN-46). A “*” symbol represents significance (*p*-value < 0.05). **Panel C.** Bar plot representation of antigenic evaluation (ELISA) of select FXIII-B cysteine mutants which showed higher intracellular retention (on confocal immunostaining) when compared to wild-type. A “*” symbol represents significance (p-value < 0.05).

Subcellular distribution of the selected FXIII-B protein cysteine mutants evaluated by confocal microscopy showed significant intracellular retention when compared with wild-type FXIII-B protein (Figure 2A,B). The retention was primarily observed in ER (C59A, C91A, C118A, C153A, C180A, and C396A) with only two mutants showing higher detectable levels in trans-Golgi body (C59A and C91A) (Figure 2C). Classically secreted proteins usually attain their primary secretable folds in the ER, after which they are transported to the Golgi apparatus to be subsequently secreted. In the event of misfolding, exposed hydrophobic patches on the unfolded protein are detected by the quality control system of the cell, and these proteins are subsequently degraded via the ubiquitin response pathway. The two mutations i.e., C59A and C91A which show Golgi retention also show high retention in the ER (Figure 2B). These cysteine mutants possibly override the ER quality control system but then are returned back by Golgi through retrograde transport and subsequently degraded [28]. The C59A and C91A mutations, therefore, show no detectable secreted protein at all (Figure 2C). The remaining mutants show accumulation in ER, but their levels in Golgi are similar to that of the wild type. This suggests that these cysteine mutants to some degree escape the ER-mediated stress response and the molecules that do make it to the Golgi are successfully secreted outside. These mutants, therefore, show low but detectable amounts of secreted protein. Clearly, disrupting different disulfide bonds affects the overall fold of the protein differently which then dictates their intracellular as well as extracellular fates. Our previous expression study on two of the reported cysteine variants also reflected this diversity, since the C25R mutant (C5R; earlier nomenclature) was observed to get strongly retained in ER and also showed no detectable secreted protein while the other C336F (C316F; earlier nomenclature) mutant was not significantly retained in either ER or Golgi and hence also showed low but detectable amount of secreted [13,14]. Another mutant, i.e., C450F (C430F; earlier nomenclature) reported and analyzed previously in BHK cell lines also showed strong retention in the ER by pulse-chase experiments and no secreted protein [6]. The C336F variant though also showed lower levels of intracellular distribution in both ER and Golgi unlike for almost all variants that we have studied which uniformly showed higher levels of intracellular retention than the wild type. As also explained in our earlier study this difference might originate from the fact that in the case of the C316F (C336F; current nomenclature used in this study), the mutation might have resulted in slower folding rates for the final protein.

### 2.3. Secreted FXIII-B Cys-Mutants Show Altered Complexation States and Possible Dimer Disruption

**Figure 3:**
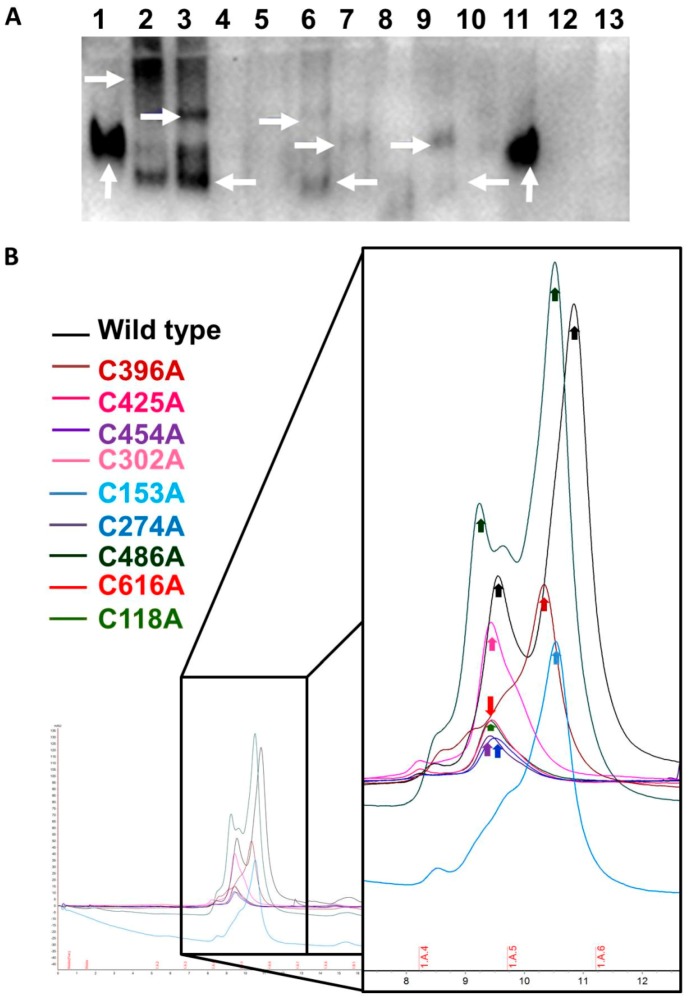
Altered complexation/Oligomerization of FXIII-B Cys mutants. **Panel A**. Western blot of rFXIII-B cysteine mutants after separation by native PAGE which were found to be secreted successfully (as detected by ELISA). Lanes: 1: Wild-Type; 2: C153A; 3: C118A; 4: C180A; 5: C267A; 6: C274A; 7: C396A; 8: C425A; 9: C454A; 10: C616A; and 11: rFXIII-B (Zedira, 75ng); 12: C302A. Vertical white arrows represent the Wild-type and rFXIII-B (Zedira GmbH, Darmstadt, Germany), whereas horizontal white arrows here indicate the diverse mobility of protein bands corresponding to FXIII-B (since the western blot has been probed with the mouse to human FXIII-B antibodies) **Panel B.** Gel-filtration chromatography of purified FXIII-B cysteine mutants. Color codes represent respective mutants indicated as inset in the figure. The x-axis denotes retention volume [ml], and the y-axis represents the amount of protein in mAU (UV-280nm).

Gel filtration runs for the recombinant wild type FXIII consistently showed two peaks, one of lower molecular weight (faster retention time) and the other of higher molecular weight (slower retention time). The high molecular weight peak with slower retention time was checked with Western blot and mass spectrometry and confirmed to contain FXIII-B subunits. No FXIII-B subunit was detected in the low molecular weight faster-retained peak. This peak, when tested with mass spectrometry, was confirmed to contain only albumin. Earlier studies with gel filtration runs conducted on FXIII-B purified from insect cell line also suggest FXIII-B to be dimeric with a single peak only although sedimentation studies contradict this evidence and instead suggest the FXIII-B be a monomer. The contradiction might reflect the flexibility of sushi domain-containing proteins to adopt different oligomeric states under different conditions as observed for CFH and that in fact for FXIII-B both monomeric and dimeric forms might be possible depending on their native physiological milieu. The Native PAGE runs for our recombinant wild type FXIII-B subunit also showed a single dimeric band. The gel filtration runs for the secreted cysteine mutants showed a different mobility pattern than the wild type. The C486A mutant showed two major peaks similar to the wild type but with faster mobility (lower retention times) and a small shoulder peak closer to the high molecular weight peak. This mutant might result in partial monomerization of the protein. Since this protein now has a free reactive cysteine, the monomeric form might interact with the albumin generating the altered peak pattern. The other mutants except for C153A and C396A majorly showed only one major peak closer to the FXIII-B subunit peak observed in the wild type run but with slightly different mobility. Mutants C153A and C396A also showed one peak, but these were closer to the albumin peak detected in the wild type run. These peaks although closer to albumin peak detected in the wild type run had clearly different retention times than the albumin peak. These mutants most likely also behave like the C486A variant, with the difference that in these mutants the dimeric peak is completely non-existent since these mutants might completely disrupt the dimer and the resulting monomers with the reactive cysteines interact with albumin (possibly by forming a disulfide bond with another free cysteine on albumin) generating these altered patterns. Native PAGE of the mutants was also on similar lines showing altered band patterns than the wild type most likely suggestive of altered complexation states with albumin (Figure 3A). High molecular weight bands were observed for C153A and C118A mutants; mutants C274A and C454A showed low molecular weight bands, as compared to wild-type, hinting towards degradation, a few mutants (C180A and C267A) showed a smeary appearance also indicating degradation of the protein upon secretion. The variants C396A and C425A showed low levels but similar mobility as the wild-type protein. The mutants C180A and C302A showed paradoxical behavior on gel filtration columns and on Native PAGE. The mutant C180A was detected on Native PAGE gels, but was not retained in the gel filtration runs. This might reflect poor stability for this mutant which got degraded before gel filtration could be conducted. The mutant C302A, on the other hand, was detected in gel filtration runs but was non-detectable in the Native PAGE. This mutant might have a completely altered fold in the dimeric state thereby evading detection from antibodies in the Native PAGE. Almost all of the proteins after purification, showed levels close to baseline in these qualitative analyses when compared to wild-type, indicating early aggregation, low shelf-life, or degradation, even after successful secretion.

### 2.4. A Filamentous Monomer FXIII-B Subunit Model with Its N and C Terminals Aligned Close to Each Other

**Figure 4:**
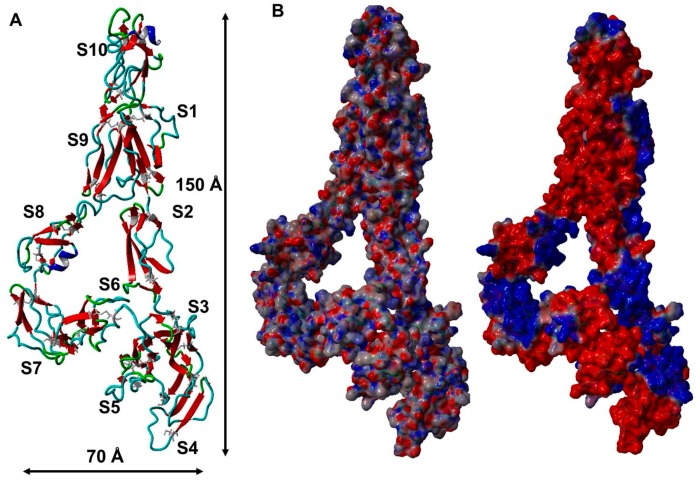
The FXIII-B subunit monomer model. **Panel A** shows the FXIII-B subunit monomer post-equilibrated model structure in ribbon format. The backbone is colored based on secondary structure. The disulfide-bonded cysteines are represented as grey colored stick forms. The individual sushi domains are numbered S1 to S10; N to C terminal. **Panel B** is the electrostatic surface representation of the FXIII-B subunit monomer model. The left side of Panel B shows the PBS styled depiction of surface electrostatics while the right side shows the PME styled depiction of surface electrostatic. Calculation and depiction for both forms of electrostatics were performed with macros embedded in YASARA. Red color indicates negative potential while blue represents positive potential.

The final model of the FXIII-B subunit post equilibration shows a filamentous structure approximately 150 Å in length and 70 Å in width (Figure 4A). All cysteines in the simulation-equilibrated and the original assembled model were in the oxidized state. The N and C terminals of the structure are observed to interact with each other with a major twist in the middle region occupied by the S3, S4, and S5 domains. This twist and the N-C-terminal interaction appear to curtail the length of the monomer which otherwise would extend much longer. The initially assembled full-length model is quite different than the MD simulation-equilibrated model (Figure S1). This is evident from the huge change in conformation/RMSD (~23 Å) during the equilibration phase of the simulation (Figure S1). It is also clear from the length of the equilibration phase (~120 ns) that the original assembled model takes a long time to adopt an energetically stable monomeric state. While the N and C terminal ends of this monomer also align next two each other in the original assembled model, it differs from the simulation-equilibrated model significantly in form with the original model being significantly shorter than the equilibrated one (Figure S2). The C terminal region showed higher flexibility than the overall protein especially the S8, S9, and S10 domains (Figure S3). The simulation-equilibrated model shows distinct electrostatic patches around the S1, S3, S4, S7, and S8 sushi domains. We repeatedly emphasize the differences between the initially assembled model and the simulation-equilibrated final model because of the dichotomy over the dimeric state of the free FXIII-B subunit in the present literature. While initially understood to be a monomer from sedimentation coefficient studies, the free FXIII-B was later suggested to be a physiological dimer based on gel filtration chromatography performed with FXIII-B subunit expressed in insect cells [17,26]. This kind of variation in the oligomeric state is also observed in the closest homolog of FXIII-B, CFH [29]. While almost double the length of FXIII-B, CFH has also been observed in different solution states to exist in different oligomeric forms (i.e., dimer or monomer) [30]. One of the reasons proposed for the variation in conformation for these sushi domain containing proteins has been the variability in surface electrostatic patches exhibited by this domain under different solution states which promotes intra-subunit or inter-subunit interactions that shift the tendency of these proteins to choose one or the other forms (i.e., monomer or dimer) [30]. The significant conformational difference between the assembled model and the simulation-equilibrated model of FXIII-B subunit suggests that this protein might exist in equilibrium between its respective states (monomer and dimer), with the local ionic environment dictating which of these states dominate.

### 2.5. The FXIII-B Subunit Disulfide Bonds Display Variability in Structural Flexibility, but Ablation of any of These Bonds Leads to a Loss in Stability

**Figure 5:**
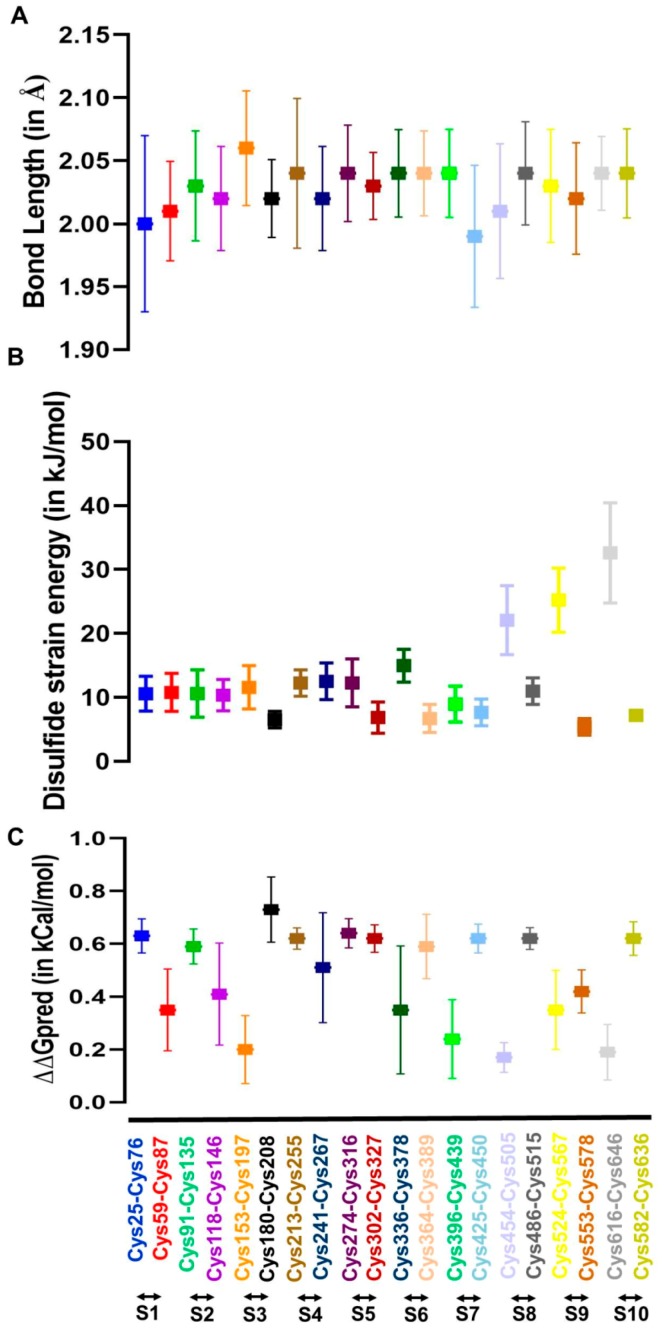
Evaluation of flexibility and stability of the disulfide bonds in the FXIII-B subunit monomer model. **Panel A** shows the variation in disulfide bond lengths during the simulation post equilibration of the FXIII-B subunit monomer model. **Panel B** shows the variation in disulfide strain energies of all disulfide bonds of the FXIII-B subunit monomer model during the MD simulation post-equilibration of the structure. **Panel C** shows the variability in free energy change when individual disulfide-bonded cysteines (the cysteines that have been mutated and expressed in this study) within the FXIII-B subunit monomer model are mutated to alanine. The evaluation was performed on several simulation trajectory structures (spaced at an interval of 20 ns) within the post-equilibrated simulation.

The disulfide bonds of the FXIII-B subunit monomer model displayed variability in bond length and dihedral energy during the production phase. The disulfide bonds showed bond length variability between 1.95–2.09 Å during their thermal motion which is typical of structural disulfide bonds [31]. The maximum bond length was observed for Cys153-Cys197 (2.06 + 0.05 Å) of S3 sushi domain during the production phase (Figure 5A). All disulfide bonds were typically right handed and left handed spiral forms which is also typical of structural disulfide bonds. The dihedral energies (or dihedral strain energies) for C-terminal disulfide bonds of sushi domains S8, S9 and S10, i.e., Cys were observed to be the highest amongst all disulfide bonds and also showed a high degree of variability (22.07 + 5.41, 25.21 + 5.01, and 30.21 + 16.26 kJ/moL, respectively) (Figure 5B). The change in free energy upon ablation of all disulfide bonds indicated a loss in stability with the highest loss in stability observed for the C180A variant present on S3 sushi domain (Figure 5C; ΔΔG = 0.73 + 0.11).

### 2.6. Disruption of Selected FXIII-B Disulfide Bonds can Affect the Rate of FXIII-Aa Generation

**Figure 6:**
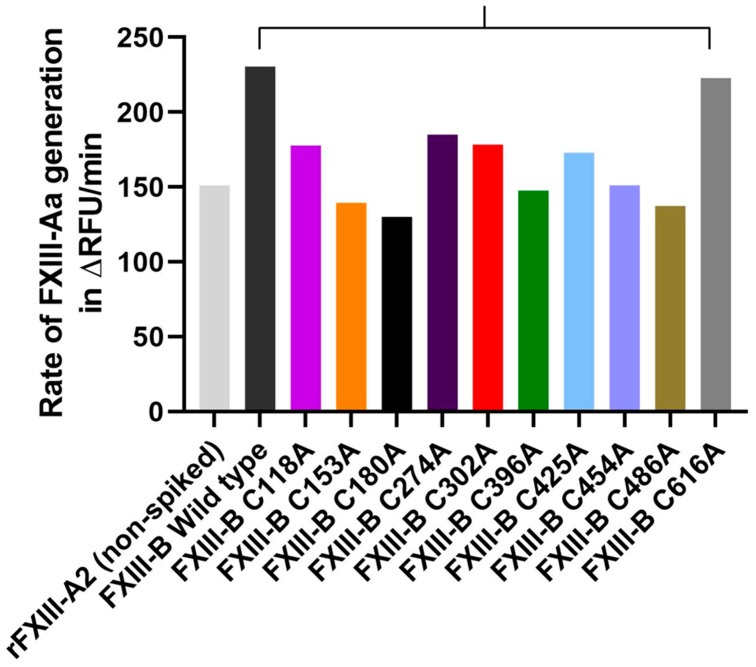
Effect of cysteine mutants on the rate of FXIII-Aa generation. This figure represents the comparative bar-plot representation of FXIII-Aa generation assay. The maximum rate of FXIII-Aa generation (µ) (in ∆RFU/min) upon spiking of FXIII-deficient plasma with 7 µg/mL rFXIII-A_2_ is represented in the x-axis, in the absence (grey bar), or presence (color-coded bars) of rFXIII-B_2_ subunits or FXIII-B cysteine mutants (10µg/mL each).

We had earlier demonstrated that spiking FXIII-B into FXIII-Aa generation assay could accelerate the generation of FXIII-Aa i.e., FXIII-A activation [2]. The recombinant wild type rFXIII-B subunit from this study also behaves similarly since we observe a jump of ~150% in the rate of FXIII-Aa generation/ FXIII-A activation when the wild type recombinant was spiked into FXIII-Aa generation assay (rate of FXIII-Aa generation in the absence of FXIII-B: 151.05 ∆RFU/min; rate of FXIII-Aa generation when spiked with 10ng/mL of purified wild-type FXIII-B: 230.06∆RFU/min) (Figure 6) [2,23,32]. When spiked with mutants C118A, C274A, and C302A a milder effect on FXIIIA activation was observed when compared to spiking with the wild type rFXIII-B. Nevertheless, these three mutants still showed the rate of activation higher than the spiked sample. Spiking with mutants C396A and C454A show the same rate of FXIII-Aa generation as observed for the non FXIII-B spiked sample suggesting that these mutations completely abolish the accelerative effect of FXIII-B on FXIII-A activation. Spiking with C616A showed almost a similar effect on FXIII-Aa generation as that of the wild type, indicating that in spite of the disruption of the disulfide bond, the regulatory (accelerative) effect on FXIII-A is retained by this protein variant. Finally spiking with three mutants C153A, C180A and C486A contribute to a lowering of the rate of FXIII-Aa generation overall, i.e., even lower than that of non FXIII-B spiked sample. These mutants might be negatively regulating the activation of FXIII-A. Since only equal absolute amounts of each mutant, as well as wild type, were spiked into the assay, the observed effects can only be attributed to the functional effect of FXIII-B on the activation of FXIII-A and not to a quantitative effect, especially for the mutants. However, a major limitation of this aspect of our study is that we were not able to repeat this study multiple numbers of times to assign significance to the differences observed. This was especially owing to the poor overall yields and the degradation tendency for the mutants. However, the wild type FXIII-B was multiply spiked in the generation assay, and the differences observed with the mutations exceeded the variability observed for the wild type rFXIII-B.

## 3. Discussion

Disulfide bonds in the protein are primarily responsible for structural stability unless the nature of these disulfides is allosteric [33]. This is the reason why substitutive mutations resulting in their disruption often result in faulty proteins complicit in pathomolecular states. However, in proteins that contain multiple disulfide bonds, the effect of a single disulfide bond disruption might vary, depending on the position of that disulfide bond in the functional hierarchy of the protein. This means essentially that in multiple disulfide bonded proteins all disulfide bonds do not carry the same functional importance. The disulfide bonds themselves stabilize the domain/part of the protein that they are part of and by extension; their importance is contingent upon the role that the particular domain might play in the protein. Some domains/part of the protein might be rendered redundant during the course of evolution thereby also rendering the importance of the disulfides that they contain useless. Proteins containing multiple repetitive domains like sushi domain are examples in which functional hierarchy/diversity within disulfide bonds might exist. The FXIII-B subunit protein which is built from ten sushi domains, each of which contains two disulfide bonds displays such diversity. Mutations on these disulfide bonds have been reported in the two forms of congenital bleeding disorders associated with mutations in the FXIII-B subunit gene, i.e., the inherited severe FXIII deficiency and the mild FXIII deficiency [13,14]. The C430F (C450F; current nomenclature used in this study) mutation was detected in a 32 year old female patient with a bleeding tendency born of a consanguineous marriage [7,11]. The plasma of this patient showed no detectable levels of FXIII-B subunit. Additionally, FXIII-A subunit concentrate, when infused into this patient showed a significantly reduced half-life. The mutation C5R (C25R; current nomenclature used in this study) was detected in a female patient who reported bleeding symptoms post abdominal surgery. This patient showed simultaneous reduction of FXIII-A and FXIII-B subunit antigen levels, as well as of FXIII activity, below 50% of the normal, which is typical of mild FXIII deficiency. The mutation C316F (C336F; current nomenclature used in this study), was detected in two individuals, one male and one female, who reported epistaxis and hematoma in the neck post brain surgery, respectively. No antigenic levels were performed for these patients, however FXIII activity was demonstrated to be in the mild-FXIII deficiency range (20–60%). Our earlier investigations had shown that these mutations, although occurring on structural disulfides will have different degrees of severity of impact on the secreted protein [13]. Our current directed mutational investigation further substantiates this fact. When we disrupt the FXIII-B disulfide bonds one at a time, the effect on the secreted protein varies from mutant to mutant. A majority (12 out of 20) of them are non-secreting mutants, the ones showing secreted amounts of protein do so in significantly diminished amounts except for two mutations C118A and C153A which show close to 50% of the wild type protein (Figure 1). However, an overwhelmingly uniform observation made for all the mutants, secreted or not was their intracellular accumulation. All mutants showed intracellular accumulation and more specifically/commonly accumulation in the ER (Figure 2). Therefore, we conclude that mutating any structural disulfide bond within FXIII-B subunit would result in a secretion deficit. The mutants that were successfully secreted were observed to show an altered complexation pattern in gel filtration runs as well as with Native PAGE. Therefore, the disruption of the specific disulfide bonds either result in alteration of FXIII-B’s physiological dimeric state or the presence of a reactive cysteine (the reduced cysteine partnering the mutated cysteine) results in the interaction of the mutant FXIII-B with other neighboring proteins (through new putative disulfide bonds). Amongst the successfully secreted mutants, half of them retained their accelerative effect of the rate of FXIII-A activation/FXIII-Aa generation (as observed for wild type FXIII-B) in spite of their low amounts (Figure 6). The fact that FXIII-B subunit can positively regulate (accelerate) the activation of FXIII-A subunit has already been reported in the recent past [2,23]. The remaining half showed either a complete abolition of this effect or a negative effect on the rate of FXIII-A activation/FXIIIAa generation. Mutating specific FXIII-B cysteine´s (check results and Figure 6) could, therefore, abolish the proteins regulatory effect on FXIII-A subunit or might even affect it negatively. However, in the context of the current experimentation performed, we cannot offer a mechanism by which these mutations might affect interaction/regulation with/of FXIII-A subunit since this would require additional quantitative binding studies. Structurally, based on our analysis of the FXIII-B monomer model, differential structural flexibility of disulfide bonds across the FXIII-B subunit is a critical factor in determining its native fold as well as its putative interactions during dimerization/interaction with other proteins. The dihedral energies of the disulfide bonds (or disulfide strain energies) were particularly on the higher end and more variable towards the C-terminal of FXIII-B subunit. Therefore, the disulfide bonds at the C-terminal of the FXIII-B subunit appear to control the overall global flexibility of FXIII-B. The FXIII-B monomer model clearly shows distinct electrostatic patches which could play an important role in its dimerization or interaction with other proteins like the FXIII-A subunit (Figure 4). The global effect of disruption of individual disulfide bonds is likely mediated by the effect that this has on a) the local structural flexibility b) neighboring electrostatic patches. Consistent with our observations made from the expression of the cysteine mutants, the in silico analysis of these disulfide bonds also shows variability in effect on overall stability (free energy; ΔΔG) (Figure 5C). To summarize, we conclude that all 20 FXIII-B subunit disulfide bonds are important to the structural stability, secretion, and function of the protein. However, these disulfide bonds do show structural and functional diversity as gauged from the variable effects we observed when we mutated them individually. In the inherited severe form of FXIII deficiency in which mutations in FXIII-B subunit are even rarer; it is difficult to conceive of detecting more cysteine mutants. Rather these cysteine mutants might exist in more preponderance in the inherited mild form of FXIII deficiency or heterozygous FXIII deficiency. Even in a heterozygous state, cysteine mutations can conceivably have a dominant negative effect, because, as observed in our study, the release of a reactive cysteine can open up the mutated variant to different interactions with another of its own subunit (during folding) or other proteins leading to change in complexation states (also observed in our study). This makes these disulfide bonds potential mutational hotspots in the mild form of this rare bleeding disorder.

## 4. Materials and Methods

### 4.1. Cell Lines and Cell Culture

The human HEK293t cell line was purchased from DSMZ German Collection of Microorganisms and Cell Cultures, (Braunschweig, Germany). All cells were cultured in high glucose DMEM (Life Technologies Europe BV, Bleiswijk, Netherlands), supplemented with 10% v/v FBS (Invitrogen), 1% v/v penicillin-streptomycin antibiotics and 0.1% v/v Fungizone (Life Technologies Europe BV, Bleiswijk, Netherlands), at 37 degrees in 5% CO2 incubator. All experiments were performed on sub-cultured cells in the logarithmic phase (below passage 20).

### 4.2. Cloning and Expression of FXIII-B Cysteine to Alanine Mutants

Human *F13B* cDNA (ORF length 1986 bp) was inserted into the cloning site of pEZ-M01 vector (used as Wild type construct for all the following experiments). Site-directed mutagenesis was performed on the aforementioned construct, using GeneArt Site-directed mutagenesis system (Life Technologies, Carlsbad, CA, USA). Mutagenesis was performed with the aim of disrupting individual cysteine bonds present in every sushi domain (FXIII-B C59A, FXIII-B C76A, FXIII-B C91A, FXIII-B C118A, FXIII-B C153A, FXIII-B C180A, FXIII-B C213A, FXIII-B C267A, FXIII-B C274A, FXIII-B C302A, FXIII-B C364A, FXIII-B C378A, FXIII-B C396A, FXIII-B C425A, FXIII-B C454A, FXIII-B C486A, FXIII-B C524A, FXIII-B C553A, FXIII-B C582A, and FXIII-B C616A). All primers were synthesized by MWG Eurofins (MWG Eurofins GmbH, Ebersberg, Germany). All plasmid construct clones were completely sequenced and verified for the correct incorporation of mutation *in-house*. Wild type *F13B*cDNA and mutated DNA were transfected into mammalian HEK293T cells for transient expression. Briefly, 2.7 × 10^5^ cells were transfected with 3 µg of plasmid DNA along with 6µl of transfection reagent Lipofectamine 2000 (Invitrogen) following the manufacturer’s protocol. The culture was harvested 48 h post-transfection. FXIII-B being a secretory protein, the culture medium (supernatant) was collected, and centrifuged at 1000 g, for 5 min at 4 degrees. Additionally, to analyze intracellular contents, cellular lysis was performed using mammalian M-PER reagent (Thermo Fischer Scientific, Rockford, IL, USA) following the manufacturer’s protocol. Cellular lysate was centrifuged at 13000 g, for 10 min at 4 degrees. Both intracellular and extracellular fractions were stored at −80 degrees until further use. The samples were verified for the antigenic presence of FXIII-B protein by ELISA and Western blot analysis.

### 4.3. Antigenic Quantification of FXIII-B Cysteine Mutants

FXIII-B antigen levels were determined, upon wild-type/mutants FXIIIB were quantified in culture supernatants (extracellular secreted FXIII-B) and cell lysates (intracellular FXIII-B) using the Technozym FXIII-B:Ag Sub ELISA kit (Technoclone GmbH, Vienna, Austria) according to the manufacturer’s instructions. The standard assay detection limit was 0.95 μg/mL; lower antigen concentrations to 0.009 μg/mL (sensitivity) were determined according to the manufacturer’s dilution protocol. FXIII-B levels from normal pooled plasma and from high/low controls from the kit were measured as controls.

### 4.4. Western Blot Analyses

For antigenic estimation in extracellularly secreted FXIII-B protein in cysteine mutants, western blot was performed using 10 µL of crude supernatant. Briefly, an equal volume of total crude protein corresponding to Wild-type (positive control), Un-transfected control (negative control), and 20 cysteine mutants were separated on 4%–16% gradient SDS-PAGE (Bio-Rad Laboratories, Hercules CA, USA). Resolved proteins were transferred to PVDF membrane at 80V for 90 min in cold-room. The membrane was blocked for 1 h at room temperature in blocking reagent (3% w/v BSA in PBS with 0.05% Tween-20). Subsequently, after a wash with PBS-Tween-20 (0.05%), the membrane was incubated for 1 h at room temperature in Primary antibody (250ng/mL) (mouse-anti human FXIII-B monoclonal antibody, in-house generated in association with Eurogentec Deutschland GmbH, Cologne, Germany) with mild shaking. After washing thrice in PBS-Tween (0.05%), the membrane was incubated for 1 h at room temperature in HRP tagged Secondary antibody (50ng/mL) (Goat Anti-mouse IgG (H+L) Secondary antibody, HRP; Thermo Fischer Scientific, Carlsbad, CA, USA). Finally, the membrane was washed thrice in PBS-Tween 20, and PBS, respectively. Chemiluminescent signal quantification, Image acquisition (ChemiDoc MP, Bio-Rad) and densitometric evaluation of signal were performed on Image lab Software (Bio-Rad) version 4.1.

### 4.5. Confocal Immunofluorescence of Expressed FXIII-B Protein and its Cysteine Mutants

The HEK293t cells, transiently transfected for expression of FXIII-B protein, were subjected to immunofluorescence analyses to evaluate intracellular trafficking of wild-type vs. cysteine mutants of rFXIII-B protein. The mutants that exhibited no extracellular secretion and high intracellular retention as compared to wild-type in ELISA assessments were tested further. Briefly, 24 h post-transfection HEK-293T cells (grown on glass coverslips) were fixed using 4% (w/v) PFA in PBS, followed by blocking with 0.1% (v/v) Triton-X 100 in PBS azide supplemented with 10% (v/v) FBS, and immunostaining with first and secondary antibodies for 2 h and 1 h, respectively. Trafficking analyses were performed via cell-specific markers; FXIII-B subunit (mouse monoclonal IgG, in-house generated in association with Eurogentec, Belgium) and the cell compartments ER (IgG rabbit polyclonal anti-calnexin; Abcam, England) and Golgi (anti-TGN46 antibody produced in rabbit; Sigma-Aldrich, Saint Louis, MO, USA) Signal detection was performed using an IgG Alexa Fluor 488 conjugated goat anti-mouse IgG (H+L) secondary antibody (Life technologies, Carlsbad, CA, USA) against the FXIII-B subunit and an IgG Alexa Flour 594 conjugated goat anti-rabbit IgG (H+L) secondary antibody (Life technologies, Carlsbad, CA, USA) against the ER and Golgi compartment. The coverslips were mounted onto microscope slides with Vectashield antifade mounting medium (Vector Labs, Burlingame, CA, USA) and analyzed with the Olympus Fluo View FV1000 or Leica SL confocal microscope. The comparative degree of co-localization for wild type versus mutants was calculated as mean Pearson’s correlation coefficient in ImageJ software. A minimum number of *n* = 10 regions of interests were extracted from the micrographs to calculate the degree of colocolization.

### 4.6. Native-PAGE-blot Analysis of Expressed FXIII-B Protein and its Cysteine Mutants

In order to track the oligomeric state of successfully secreted FXIII-B cysteine mutants (detected by conventional western blot and ELISA), non-denaturing Native-PAGE was performed (Life Technologies Europe BV, Bleiswijk, Netherlands) as per manufacturer’s guidelines (only for the mutants reflecting wider bands in Western blots above) (Figure 3A). Briefly, samples were prepared using the BN Sample buffer (Life Technologies Europe BV, Bleiswijk, Netherlands ). After electrophoretic separation, the gel was placed in 2X NuPAGE Transfer buffer (Life Technologies, Carlsbad, CA, USA) for 10 min at RT. Subsequently, protein transfer to PVDF membrane was performed at 60V for 90 min in cold-room, followed by blotting (as Western blot procedure mentioned above).

### 4.7. Purification of Wild Type FXIII-B and its Cysteine Mutants

Secreted protein harvested post transfection of HEK293T cells, was concentrated 15-20 times, using Amicon ultra-filters (Cut-off 30,000Da, Merck, Darmstadt, Germany) and was subjected to immuno-affinity based purification using the Thermo Scientific Pierce Co-IP kit (Pierce Biotechnology, Rockford, IL, USA) following the manufacturer’s protocol. Briefly, mouse monoclonal antibodies against human FXIII-B (generated in-house in association with Eurogentec Deutschland GmbH, Cologne, Germany) were immobilized to Amino-Link plus coupling resin, for 2 h at room temperature. The resin was then washed and incubated with transfected HEK293T cellular medium concentrates (with the detected antigenic presence of FXIII-B protein (Figure 3A)) overnight in cold-room. Next day, the resin was washed, and protein bound to anti-FXIII-B antibody was eluted. Eluted protein was further subjected to gel filtration chromatography, to ensure its purity and to characterize the oligomeric association of secreted protein upon mutation in comparison to wild-type (Figure 3B). Gel filtration chromatography was performed on Äkta Pure protein purifier system, using Superdex® 200 Increase column (GE healthcare UK Ltd, England, UK). All purifications were performed in a cold room, on a column pre-equilibrated with PBS, pH 7.4 (flow rate 400µL/min). The comparative oligomeric state of wild-type versus mutant proteins was calculated on the basis of peak retention time for each mutant vs. wild-type FXIII-B.

### 4.8. In Silico Analysis of FXIII-B Subunit Disulfide Bonds

In the absence of a biophysical structure for the FXIII-B subunit, we generated a full-length monomer model of the FXIII-B subunit. The model was built by assembling previously reported high quality threaded models of all ten FXIII-B subunit sushi domains on the AIDA domain assembly server (http://aida.godziklab.org/) (accessed on November 19^th^, 2018). in default mode, i.e., without any constraint [16,34]. The model was initially subjected to 500 ps of refinement MD simulation using the macro md_refine embedded in YASARA [35,36]. This macro uses YAMBER3 force field parameters in YASARA in order to remove steric clashes and improve rotamer geometry [37]. The structure with the lowest energy in the simulation trajectory was chosen for conducting further simulations. This structure was then subjected to all-atom unrestrained MD simulation using the md_sim macro embedded in YASARA. Briefly, a simulation cell with periodic boundaries and 20 Å minimum distances to protein atoms was employed with explicit solvent. The AMBER03 force field, NPT ensemble was used with long range PME potential and a cut-off of 7.86 Å [38]. Hydrogen bond networks were optimized using the method of Hooft and co-workers [39]. The simulation cell was filled with water at a density of 0.997 g/mL and a maximum sum of all bumps per water of 1.0 Å. The simulation cell net charge was neutralized with a final 0.9% (wt/vol) NaCl concentration. The entire system was energy minimized by steepest descent to remove conformation stress within the structure, followed by simulated annealing minimization until convergence was achieved. The MD simulation was performed at a temperature of 298 K. Simulation was run for ~500 ns (including the time needed to equilibrate). Electrostatic surface potential was calculated and graphically depicted using the Adaptive Poisson-Boltzmann Solver integrated within YASARA [35]. The structural flexibility of the FXIII-B subunit disulfide bonds in the monomer model was analyzed on simulation trajectory snapshots captured per 250 ps in the production phase (i.e., post-equilibration). Each of these snapshots was converted to PDB format before submission to the Disulfide Bond Dihedral Angle Energy Server (https://services.mbi.ucla.edu/disulfide/) (accessed on February 7th, 2019). for evaluation of the disulfide bonds. This server calculates the dihedral angles of all disulfide bonds found in the uploaded structure PDB file. Based on these angles, dihedral energy (disulfide strain energy) is calculated according to an empirical formula first defined by Katz et al. [40]. In addition to disulfide strain energies, disulfide bond length variations for all uploaded structures were also evaluated on this server. The change in free energy (i.e., loss or gain of stability) for each disulfide bond was calculated on the MAESTROweb server (https://biwww.che.sbg.ac.at/maestro/web/maestro) ((accessed on February 7th, 2019). ) by uploading the PDB file of the final simulation snapshot in the production phase (i.e., equilibrated structure) [41]. This server predicts the change in free energy (ΔΔG) values along with a corresponding prediction quality measure for a single or list of mutations corresponding to the uploaded structure is specified. Positive ΔΔG values indicate loss of stability, while negative indicate a gain of stability. The prediction quality measure varies between 0 and 1, with values approaching 1 indicating a reliable prediction.

### 4.9. Effect of Spiking Wild Type FXIII-B and Its Cysteine Variants into the FXIII-Aa Generation Assay

FXIII-Aa generation was triggered by tissue factor/phospholipids (TF/PL), and FXIII-A isopeptidase activity was measured using the fluorogenic substrate A101 (Zedira GmbH, Darmstadt, Germany) in a Safire microtiter plate reader (Tecan, Crailsheim, Germany). Twenty microliters FXIII-deficient plasma (deficient for FXIII-A_2_ and FXIII-B_2_; Haemochrom Diagnostica GmbH, Essen, Germany) spiked with rFXIII-A_2_ (7 µg/mL), and purified rFXIII-B_2_-mutants (10 µg/mL) were incubated with 55 μL reagent solution (5 μL 100 mM glycine methyl ester, 5 μL 2 mM fluorogenic FXIII-A substrate, 10 µl phospholipids (Rossix, Mölndal, Sweden) diluted 1:10 in HBS, and 35 μL HBS (20 mM Hepes, 150 mM NaCl)/0.1% serum albumin pH 7.5. After pre-incubation of the mixture for 5 min, the reaction was started with 20 μL Innovin (recombinant TF, Dade Behring, Marburg, Germany) 1:1400 diluted in HBS, 50 mM CaCl_2_ pH 7.5. Fluorescence was measured over 1 h at λ ex = 330 nm and λ em = 430 nm in kinetic mode two-times per minute. Data were analyzed based on growth-curve analyses, with the slope of the curve (µ) representing the growth rate [32]. Data were fitted using R-package “grofit”, based on the dose-response relationship [42]. Non-parametric spline estimation was done to fit the data, and to obtain characteristic parameters lag phase tlag, maximal growth rate µ, Area under curve A, and maximal time to peak tmax) derived from a single growth curve.

## 5. Statistical Analyses

Comparisons of means were performed using a non-parametric Mann-Whitney’s t-test. All statistical analyses were performed in Graphpad Prism (Version 8.0.2). Details for curve fitting for FXIII-Aa generation assay are provided under the method section of FXIII-Aa generation assay.

## Supplementary Materials

Supplementary materials can be found at https://www.mdpi.com/1422-0067/20/8/1956/s1.
