# Supplementary material for: Disruption of Structural Disulfides of Coagulation FXIII-B Subunit; Functional Implications for a Rare Bleeding Disorder
Source: Int J Mol Sci. 2019 Apr 22;20(8):1956. doi: 10.3390/ijms20081956 (PMC6514982; doi:10.3390/ijms20081956)

Supplementary file

Disruption of structural disulfides of Coagulation FXIII-B subunit; functional implications for a rare bleeding disorder

Sneha Singh [1], Mohammad Suhail Akhter [1,2], Johannes Dodt [3], Amit Sharma [1,4], Senthilvelrajan Kaniyappan [5], Hamideh Yadegari [1], Vytatus Ivaskevicius [1], Johannes Oldenburg [1] and Arijit Biswas [1,*]

1 Institute of Experimental Haematology and Transfusion Medicine, University Clinic Bonn, 53127 Bonn, Germany

2 College of Applied medical Sciences, Jazan University,Jizan, Saudi Arabia.

3 Paul-Ehrlich Institute, Langen. Germany.

4 Department of Hematology, All India Institute of medical Sciences, New Delhi, India

5 DZNE, German Center for Neurodegenerative Diseases, Bonn, Germany

* Correspondence: PD Dr. Arijit Biswas; arijit.biswas@ukb.uni-bonn.de; Tel.: +49-228-287-19428

Figure S1. The RMSD graph for MD simulation-equilibration run of the FXIII-B subunit model. The region under blue shade is the production phase representing post equilibration simulation run.

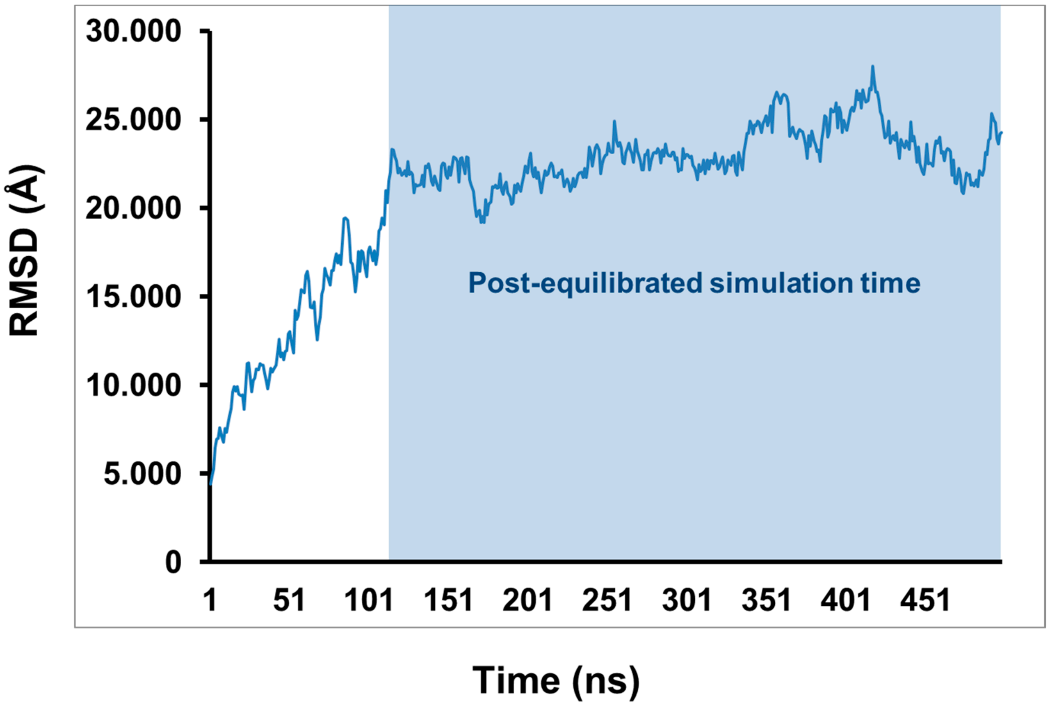

Figure S2. Difference in structures of the assembled model and the simulation equilibrated final model.

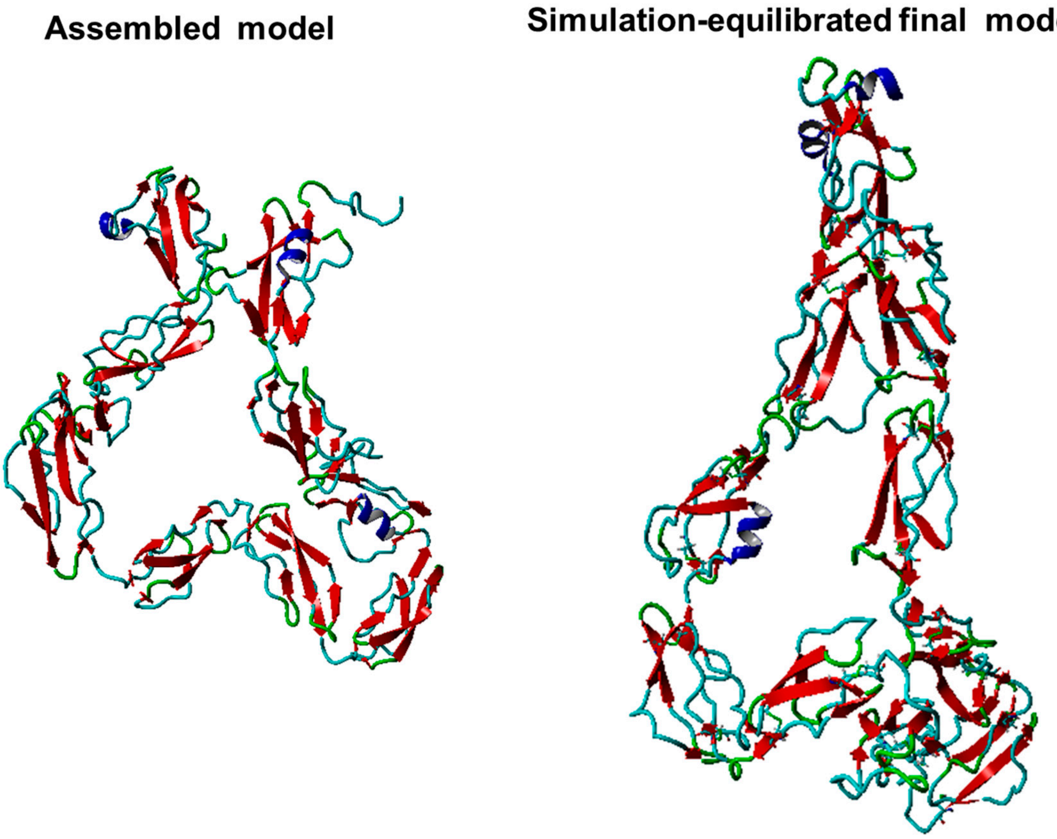

Figure S3. Flexibility/ RMSF graph for the FXIII-B subunit monomer model simulation.

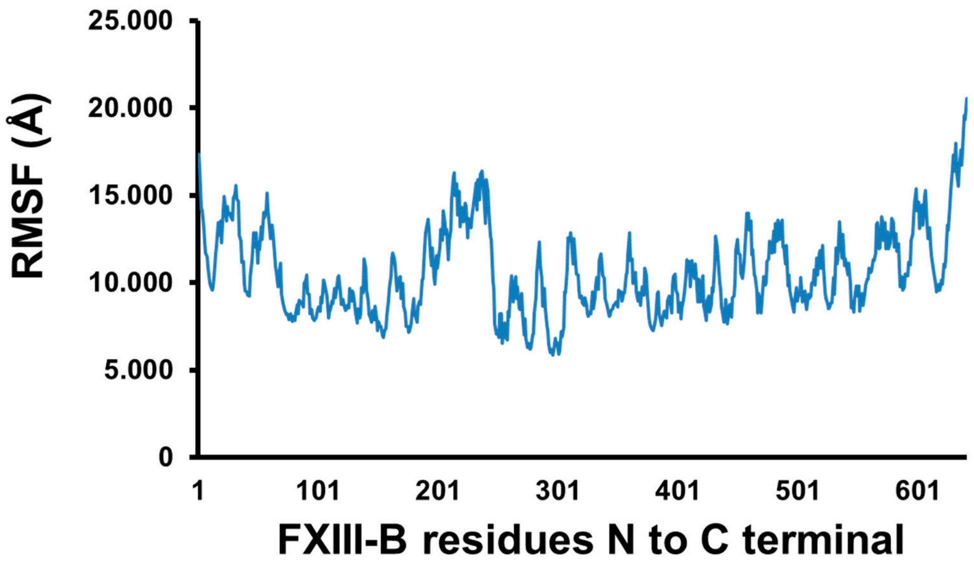

Supplement: Supplementary file 1 [file ijms-20-01956-s001.pdf]
